# The Exocyst Complex Regulates Free Fatty Acid Uptake by Adipocytes

**DOI:** 10.1371/journal.pone.0120289

**Published:** 2015-03-13

**Authors:** Mayumi Inoue, Takeshi Akama, Yibin Jiang, Tae-Hwa Chun

**Affiliations:** 1 Division of Metabolism, Endocrinology & Diabetes (MEND), Department of Internal Medicine, University of Michigan Medical School, Ann Arbor, MI, United States of America; 2 Biointerfaces Institute, University of Michigan, Ann Arbor, MI, United States of America; Tohoku University, JAPAN

## Abstract

The exocyst is an octameric molecular complex that drives vesicle trafficking in adipocytes, a rate-limiting step in insulin-dependent glucose uptake. This study assessed the role of the exocyst complex in regulating free fatty acid (FFA) uptake by adipocytes. Upon differentiating into adipocytes, 3T3-L1 cells acquire the ability to incorporate extracellular FFAs in an insulin-dependent manner. A kinetic assay using fluoresceinated FFA (C12 dodecanoic acid) uptake allows the real-time monitoring of FFA internalization by adipocytes. The insulin-dependent uptake of C12 dodecanoic acid by 3T3-L1 adipocytes is mediated by Akt and phosphatidylinositol 3 (PI3)-kinase. Gene silencing of the exocyst components Exo70 and Sec8 significantly reduced insulin-dependent FFA uptake by adipocytes. Consistent with the roles played by Exo70 and Sec8 in FFA uptake, mCherry-tagged Exo70 and HA-tagged Sec8 partially colocalize with lipid droplets within adipocytes, suggesting their active roles in the development of lipid droplets. Tubulin polymerization was also found to regulate FFA uptake in collaboration with the exocyst complex. This study demonstrates a novel role played by the exocyst complex in the regulation of FFA uptake by adipocytes.

## Introduction

Dietary lipids constitute approximately 40% of caloric intake in modern human diet [1]. Free fatty acids (FFAs) not only serve as important energy source for ATP synthesis but also regulate intracellular signaling and transcription [2]. FFAs in circulation are rapidly incorporated into adipocytes, hepatocytes, and cardiac myocytes [3]. Circulating FFA levels are regulated not only by dietary FFA intake but by hormones and sympathetic tones [4]. Dysregulated FFA handling may contribute to impaired glucose metabolism found in obese and diabetic subjects [5,6]. Therefore, defining the molecular and cellular mechanisms that regulate FFA uptake should help us better understand the pathogenesis of obesity and insulin resistance. A cohort of receptors and transporters, e.g., CD36 and fatty acid transporters (FATP) 1–4, have been shown to regulate adipocyte FFA uptake [7–12]. The plasma membrane-mediated flip-flop mechanism of FFA translocation is also suggested to regulate cellular FFA uptake [13,14]. However, the role of intracellular vesicle trafficking in the regulation of FFA uptake has not been examined to this date.

The exocyst is a large protein complex composed of Sec3 (Exoc1), Sec5 (Exoc2), Sec6 (Exoc3), Sec8 (Exoc4), Sec10 (Exoc5), Sec15 (Exoc6), Exo70 (Exoc7), and Exo84 (Exoc8). The exocyst complex was initially discovered in yeast as a molecular machinery that regulates the exocytosis of secretory vesicles [15]. In mammalian cells, the exocyst complex promotes the translocation of glucose transporter type 4 (GLUT4) from the intracellular compartment to the plasma membrane [16–18]. Diverse biological roles of the exocyst complex have been described in different cell types including insulin secretion from pancreatic beta-cells [19,20], the trafficking of neurotransmitter receptors in synaptic terminals [21], and the membrane-localization of a matrix metalloproteinase (MMP) in cancer cells [22]. In adipocytes, however, the metabolic role played by the exocyst complex beyond insulin-dependent glucose uptake has not been fully explored.

In this study, we have identified a new role for the exocyst complex in the regulation of FFA uptake by adipocytes. Our findings may shed new light on the molecular mechanism underlying FFA handling in health and diseases.

## Materials and Methods

### Cell culture and adipocyte differentiation

The 3T3-L1 cells (ATCC, CL-173) were maintained in DMEM, 25 mM glucose (Gibco) with 10% new born calf serum (NCS, Hyclone) in a 5% CO_2_ incubator at 37°C. The adipocyte differentiation of 3T3-L1 cells was induced by changing media to DMEM, 25 mM glucose with 10% fetal bovine serum (Hyclone) containing a differentiation mix (100 nM insulin, 0.25 μM dexamethasone, and 0.5 mM 3-isobutyl-1-methyxanthine, all from Sigma-Aldrich)[23]. Three days after the induction of adipogenesis, 3T3-L1 adipocytes were cultured in an optical 96-well plates with DMEM supplemented with 25 mM glucose, 100 nM insulin, and 10% FBS.

### Free fatty acid uptake assay

Lipid uptake assay was performed using QBT Fatty Acid Transporter Assay Kit (Molecular Devices) according to the manufacturer’s instruction [24]. About 50,000 cells/well/100 μL 3T3-L1 adipocytes were plated onto an optical 96 well plate (Fischer Scientific) and centrifuged at 1000 rpm for 5 min. After overnight incubation at 37°C with 5% CO_2_, media were changed to serum-free DMEM of high-glucose (25 mM) or low-glucose concentration (5.5 mM), and incubated for additional 1 hour. Cells were stimulated with 10 nM insulin for 30min in 1x assay buffer (1x Hank’s balanced salt solution with 20 mM HEPES and 0.2% fatty acid-free BSA) before the assay, then the fluorescent emission from each well was measured immediately after adding QBT Fatty Acid Uptake solution [24]. The unquenched emission of intracellular BODIPY-dodecanoic acid was measured in a Victor II Multilevel Plate Reader (PerkinElmer) or Synergy Neo Multi-Mode Reader (Bio-Tek) in real time up to 3,000 seconds (λex = 480nm and λem = 515nm).

### Inhibitors

A phosphoinositide-3-kinase (PI-3K) inhibitor (Wortmannin), MEK inhibitor (U0126), mTOR inhibitor (rapamycin), Akt1/2 kinase inhibitor (Akt1/2I), and nocodazole were obtained from Sigma-Aldrich (St. Louis, MO).

### Stealth RNAi transfection into adipocytes

3T3-L1 adipocytes were transfected with stealth RNA interference (RNAi) duplexes (Invitrogen) using electroporation as described before [17]. Adipocytes at day 2 post-differentiation were detached from culture dishes with 0.25% trypsin, washed twice, and suspended in phosphate-buffered saline (PBS). Approximately 5 x 10^6^ adipocytes were mixed with 100 nM RNAi duplexes and the electroporation was performed at 0.16 kV, 960 F with a Gene Pulser II (Bio-Rad). After electroporation, cells were incubated in DMEM with 10% FBS for 10 min at 37°C in 5% CO_2_ incubator for recovery. The sequences of stealth RNAi used were the following: *Exo70*: GCA GCU GGC UAA AGG UGA CUG ACU A, *Exo70* control: GCA CGG UAA AUG UGG GUC AAC GCU A, *Exo70* oligo #2: GCG CCA UCU UCC UAC ACA ACA ACU A, Exo70 oligo #2 control: GCG UCU AUC CUC ACA ACA AAC CCU A, *Sec8*: GGA GAU UGA ACA UGC CCU GGG ACU U, *Sec8* control: GGA GUU CAA GUA CCC GGU AGG ACU U. The effectiveness of these Exo70 and Sec8 siRNA oligos was verified as described before [17].

### Mice

Two 6-week-old C57BL/6J male mice were purchased from The Jackson Laboratory and the inguinal fat pads were isolated for cDNA cloning of mouse Exo70. Mice were euthanized with isoflurane overdose and the euthanasia was confirmed with bilateral thoracotomy. All animal procedures were approved by University Committee on Use and Care of Animals (UCUCA) of the University of Michigan.

### Intracellular localization of Exo70 and lipid droplets

The mouse Exo70 cDNA was obtained from mouse (C57BL/6J) inguinal adipose tissues with RT-PCR. The mouse Exo70 cDNA was cloned into pmCherry-C1 vector (Clontech). HA-tagged Sec8 expression vector was previously described and validated [17]. The pmCherry-Exo70 construct or HA-Sec8 was transfected into 3T3-L1 adipocytes with electroporation as described above. 48 hours after transfection, adipocytes were incubated with BODIPY 493/503 (Life Technologies) for 30 minutes and fixed in 4% paraformaldehyde in PBS. Immunofluorescent staining of HA-Sec8 was performed as described [17, 23]. Nikon A1Rsi inverted confocal laser scanning microscope with 60x/1.2 NA Plan Apochromat objective lens was used to determine the intracellular localization of mCherry-Exo70 (red, 595 nm) in relation to lipid droplets (green, 495 nm). Using sequential scanning, the lack of fluorescent bleed-through between scanned images was confirmed. Colocalization of immunofluorescence signals was assessed with ImageJ (NIH) using the plug-in Colocalization_Indices.java [25], which determines the colocalization of green and red signals using Pearson’s correlation coefficient [26] and Manders’ overlap coefficient [27].

### Statistical data analysis

FFA uptake data were analyzed with area under curves and multiple t-tests and two-way ANOVA for time-dependent FFA uptake between samples. P-value <0.05 was considered as significant.

## Results

### Real-time monitoring of FFA uptake by differentiated adipocytes

**Fig 1:**
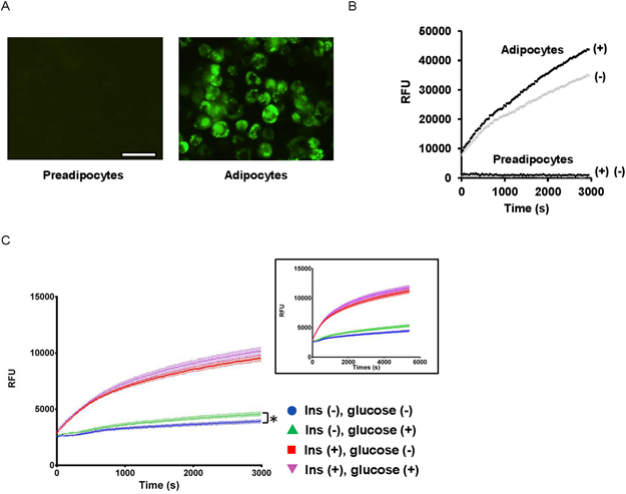
FFA uptake by differentiated adipocytes. (A) The uptake of BODIPY-C12 FA by preadipocytes and adipocytes were observed under a fluorescent microscope. Scale = 50 μm. (B) Time-dependent BODIPY-C12 FA uptake by adipocytes. Intracellular emission of BODIPY-C12 FA was monitored every 20 s up to 3,000 s in adipocytes and preadipocytes in the presence and absence of insulin (100 nM). Insulin-treated (+) and untreated (-). (C) The rate of FFA uptake was assessed with the adipocytes cultured in the presence and absence of 25 mM glucose. Blue circle (no insulin, no glucose), red square (with insulin, no glucose), green triangle (no insulin, with glucose), purple reverse triangle (with insulin, with glucose). Inset shows FFA uptake with an extended assay time. At 6,000 s, insulin-dependent FFA uptake shows the saturation of FFA uptake-dependent fluorescent signals. *P < 0.05.

To assess the FFA uptake by adipocytes, we examined the cellular uptake of the fluorescently-labeled medium-chain FFA dodecanoic acid (BODIPY-C12-FA) [24,28]. The BODIPY-C12-FA was quenched in culture media as indicated by the lack of fluorescent emission from the extracellular space; however, once C12-FFAs were incorporated into the cytoplasm of adipocytes, the quenchers were dissociated and intracellular fluorescent emission was observed [10]. While undifferentiated 3T3-L1 preadipocytes did not display any detectable fluorescent emission, differentiated adipocytes displayed a time-dependent and robust accumulation of BODIPY-C12-FA in the form of lipid droplets within adipocytes (Fig. 1A, 1B), which was consistent with previously reported observations [12,24].

To determine the kinetics of FFA uptake in real time, the fluorescent emission from unquenched fluoresceinated C12-FA was monitored using an automated fluorescent reader. When the assay time was extended to 6,000 s, the insulin-dependent increase in FFA uptake showed the signs of signal saturation (Fig. 1C, inset). The fitting to a saturation curve suggests that the time to reach the half the maximal fluorescent intensity is between 800 and 1,300 s. Therefore, following experiments were performed with assay time up to 3,000 s. The time-dependent increase of fluorescent emission following the intracellular uptake of BODIPY-C12-FA was observed only in differentiated adipocytes but not in preadipocytes (Fig. 1B), suggesting that FFA uptake is a cellular process newly acquired during adipocyte differentiation. The time course of insulin-dependent FFA uptake demonstrated that adipocyte FFA uptake begins in a liner fashion immediately after exogenous FFA are added and increases in a time-dependent manner. This adipocyte-dependent FFA uptake was increased by more than 30% in the presence of 100 nM insulin, but the effect of insulin varied depending on the degree of adipocyte differentiation (Fig. 1B). We speculated that the presence of glucose in extracellular compartment may accelerate insulin-dependent FFA uptake of adipocytes by providing glycerol [29]. To test this possibility, we assessed the FFA uptake by adipocytes cultured in the presence and absence of extracellular glucose. Basal FFA uptake was significantly higher in adipocytes cultured in DMEM with 25 mM glucose than those cultured in DMEM without glucose, albeit with a minimal effect (area under curve, AUC, no glucose, 1.01x10^7^ vs. with glucose, 1.14x10^7^, *P* = 0.017 at *t* = 3,000 s); however, under an insulin-stimulated condition, adipocyte FFA uptake did not significantly differ with and without glucose (AUC, no glucose, 2.21x10^7^; with glucose, 2.32x10^7^, *P* = 0.21 at *t* = 3,000 s, Fig. 1C). These results suggest that insulin-dependent FFA uptake in adipocyte is not significantly altered by the presence of extracellular glucose and that insulin directly regulates FFA uptake by adipocytes.

### C-12 FFA uptake by adipocytes is regulated by Phosoinositide (PI) 3-kinase and Akt

**Fig 2:**
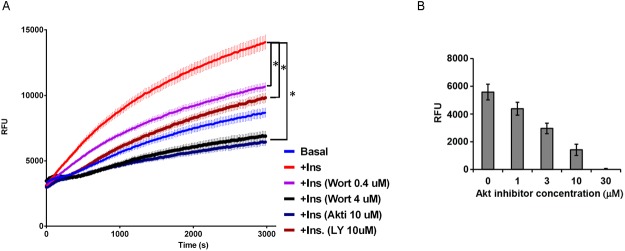
Akt- and PI3 kinase-dependent FFA uptake of adipocytes. (A) Adipocytes were pretreated with kinase inhibitors, then stimulated with 100 nM insulin for 30 minutes before FFA uptake assay. Blue, basal FFA uptake without insulin; red, with insulin; purple and black, pretreated with Wortmaninn 0.4 μM and 4 μM; navy blue, AktI 10 μM; brown, LY294002 10 μM. N = 5 each. * *P* <0.01 (B) Dose-dependent suppression of insulin-dependent FFA uptake by Akt inhibitor. FFA uptake induced by 100 nM insulin with the subtraction of basal FFA uptake. N = 3 each.

We explored the signaling pathways that are required for adipocyte FFA uptake using the kinetic assay of FFA uptake. While adipocytes were able to incorporate FFA without insulin, treatment with 100 nM insulin for 30 minutes significantly increased FFA uptake (AUC, control, 1.90 x 10^7^; insulin treated, 2.96 x 10^7^, *P* = 3.0 x 10^-5^ at *t* = 3,000 s, Fig. 2A). The inhibition of PI3-kinase activity with Wortmannin (0.4 μM) caused a substantial reduction in basal FFA uptake (AUC, 1.45 x 10^7^, *P* = 0.0008 compared to control without insulin at t = 3,000 s) as well as in insulin-stimulated FFA uptake (AUC, 2.3 x 10^7^, *P* = 0.0006 compared to control with insulin at *t* = 3,000 s) (Fig. 2A).

These findings were replicated with another PI3-kinase inhibitor, LY294002 (10 μM) (AUC, basal condition, 1.38 x 10^7^; insulin-stimulated condition, 2.07 x 10^7^, *P* = 4.2 x 10^-5^ and 0.00017, compared to controls with and without insulin at *t* = 3,000 s). An increased dose of Wortmannin (4 μM) completely blocked the insulin-induced increase of FFA uptake (Fig. 2A, AUC 1.59 x 10^7^, *P* = 4.2 x 10^-6^). Downstream of PI3 kinase, Akt2 plays a dominant role in adipose tissue development and function [30,31]. When adipocytes were treated with Akt inhibitor (Akt1/2I; 10 μM), FFA uptake by adipocytes was markedly suppressed to a degree similar to that observed with 4 μM Wortmannin (Fig. 2A, AUC 1.51 x 10^7^, *P* = 1.6 x 10^-6^ compared to insulin-treated control at *t* = 3,000 s; *P* = 0.34 between Wortmannin 4 μM and Akt1/2I 10 μM). Two-way ANOVA confirmed the time-dependent (P<0.0001) and inhibitor-dependent (P<0.0001) regulation of FFA uptake. The suppressive effect of Akt inhibitor on insulin-dependent FFA uptake was found to be dose-dependent (Fig. 2B).

### The exocyst complex partially colocalizes with lipid droplets

**Fig 3:**
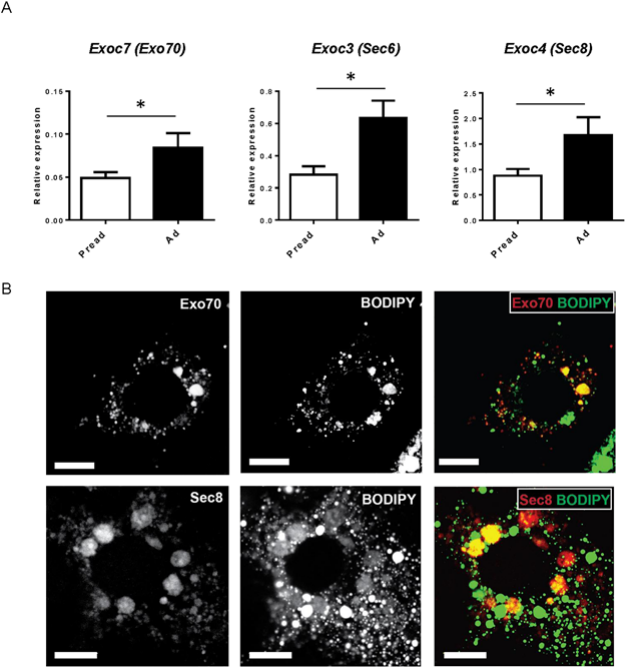
The expression and localization of the exocyst complex in adipocytes. (A) The mRNA expression of Exo70, Sec6, and Sec8 before and after 3T3-L1 adipocyte differentiation. Pread: 3T1-L1 preadipocytes, Ad: 3T3-L1 adipocytes. (B) Upper, intracellular localization of mCherry-Exo70 (red) and lipid droplets stained with BODIPY 493/503 (green); lower, HA-Sec8 (red) and lipid droplets (green). Scale = 10 μm.

The exocyst complex plays a major role in the regulation of insulin-dependent glucose uptake [16]. We hypothesized that exocyst-dependent vesicular trafficking may play a role in FFA uptake by adipocytes. When we examined the expression of the exocyst components before and after adipocyte differentiation, we observed the mRNA expressions of Exo70, Sec6, and Sec8 were significantly higher in differentiated adipocytes than in preadipocytes (Fig. 3A).

Next, we examined the localization of transfected mCherry-Exo70 and HA-Sec8 within adipocytes. The mCherry-Exo70 signals were found in intracellular vesicular structures (Fig. 3B) and were partially co-localized with intracellular lipid droplets (Fig. 3B, colocalization Pearson’s coefficient, 0.45±0.09; Manders’ overlap coefficient, 0.63±0.06, n = 7, see S1 Fig.). Similarly, HA-Sec8 partially co-localized with lipid droplets (Fig. 3B, Pearson’s coefficient, 0.34±0.06; Manders’ overlap coefficient, 0.64±0.08, n = 3).

**Fig 4:**
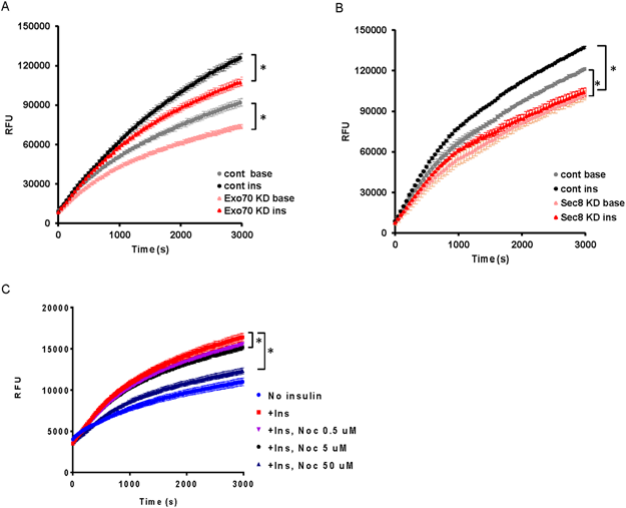
The exocyst complex mediates insulin-induced FFA uptake in adipocytes. (A) Exo70 gene expression was suppressed (Exo70 KD) with siRNA and FFA uptake by adipocytes was assessed. Gray circle (control siRNA, no insulin), black circle (control siRNA, with 100 nM insulin), yellow triangle (Exo70 siRNA, no insulin), red triangle (Exo70 siRNA, 100 nM insulin). (B) Sec8 gene expression was silenced (Sec8 KD) and FFA uptake by adipocytes in the presence and absence of insulin was determined. Gray circle (control siRNA, no insulin), black circle (control siRNA, with 100 nM insulin), yellow triangle (Sec8 siRNA, no insulin), red triangle (Sec8 siRNA, 100 nM insulin). (C) The effect of nocodazole (Noc) treatment on FFA uptake of adipocytes. Mean ± SEM. N = 4. *P < 0.05.

We next aimed to determine the effect of Exo70 and Sec8 gene silencing on adipocyte FFA uptake. Gene silencing of Exo70 significantly reduced FFA uptake under both basal and insulin-treated conditions (AUC, baseline with control oligo, 3.6x10^7^, with Exo70 siRNA, 3.1x10^7^, *P* = 0.001 at *t* = 3,000 s; under insulin treatment, control oligo, 3.7x10^7^; Exo70 siRNA, 3.3 x 10^7^, *P* = 0.01 at *t* = 3,000 s) (Fig. 4A).

Gene silencing of Sec8 also significantly suppressed basal and insulin-dependent FFA uptake by adipocytes (AUC, 3.1x10^7^ versus 2.7x10^7^ without insulin, *P* = 0.03 at *t* = 3,000 s; 3.5x10^7^ versus 2.9x10^7^ under insulin treatment, *P* = 0.01 at *t* = 3,000 s) (Fig. 4B). The intracellular localization of the exocyst complex is known to be regulated through tubulin polymerization [32]. We therefore examined the role of microtubules in FFA uptake by disrupting tubulin polymerization using nocodazole. Treatment of adipocytes with nocodazole for 4 hours demonstrated a dose-dependent inhibition of insulin-stimulated FFA uptake (AUC, insulin-treated control, 7.9 x10^-7^, +nocodazole 0.5 μM, 7.5 x 10^-7^, *P* = 0.11; 5 μM, 7.3x 10^-7^, *P* = 0.03; 50 μM 6.2 x 10^-7^, *P* = 0.0001) (Fig. 4C).

## Discussion

**Fig 5:**
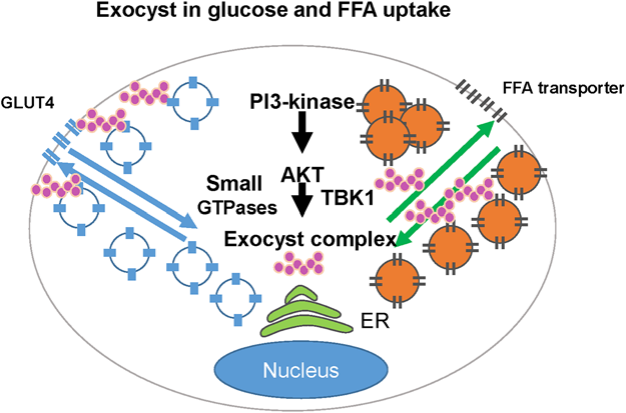
Schema of exocyst-dependent glucose uptake and FFA uptake. The translocation/fusion of Glut4-containing vesicles and FFA uptake are both regulated by the exocyst complex downstream of PI3-kinase and Akt. The exocyst functions in concert with other modifiers such as small GTPases, TBK1, and SNARE proteins that together coordinate adipocyte glucose uptake and FFA uptake.

The elevation of plasma FFA concentration is one of the key clinical findings observed in the patients with obesity and diabetes [33]. The adipose tissue is the largest insulin-sensitive organ in the body which regulates the circulating level of FFA. Adipocyte size is closely correlated with adipocyte function, insulin sensitivity, and metabolism in obesity [34]. While the exocyst complex is known to regulate the trafficking/docking of an insulin-sensitive glucose transporter, GLUT4, in adipocytes [16,17], the role of this octameric protein complex in the regulation of FFA metabolism had not been explored to this date. Our findings demonstrate for the first time that insulin-simulated FFA uptake by adipocytes is regulated through the exocyst complex (Fig. 5).

Akt2 plays a dominant role in insulin-dependent glucose metabolism as well as adipose tissue development [30,31]. Insulin-dependent FFA uptake and glucose uptake are both regulated by Akt and the exocyst complex in adipocytes (Fig. 5). It is unclear, however, how the exocyst complex coordinates GLUT4 translocation/fusion and FFA uptake in adipocytes at the same time. Our FFA uptake assay with and without extracellular glucose suggest that active glucose transport under insulin-stimulated condition does not significantly interfere with insulin-dependent FFA uptake. Therefore, partial redistribution of Exo70 to the plasma membrane necessary for GLUT4 trafficking [35] might not significantly interfere with insulin-dependent FFA uptake (Fig. 5). The mechanism by which the exocyst complex regulates FFA uptake is unclear from this study. The exocyst may regulate 1) FFA uptake at plasma membrane, 2) the trafficking of FFA to lipid droplets, or 3) the fusion and maturation of lipid droplets. SNAP23, a t-SNARE protein [36,37], mediates the fusion of lipid droplets [38]. Exo70 interacts with Snapin, which binds to SNAP23 [39]. The crosstalk between SNAP23 and Exo70 may directly or indirectly regulate FFA uptake in adipocytes. Additional modifiers, such as small GTPases [16,40], may also determine the coordinated balance between glucose and FFA utilization through the exocyst complex in a cell- and tissue-specific manner (Fig. 5). RalA was found to be a key component of lipid droplets [41]. The proteome analyses in drosophila have demonstrated the presence of Rab8 and Exo70 in lipid droplets [42,43]. Therefore, Exo70 and other members of the exocyst complex may regulate the trafficking and the fusion/maturation of lipid droplets in concert with small GTPases, such as RalA and Rab8.

The components of the exocyst complex are phosphorylated in response to insulin, EGF1, and ERK [44–46]. The role of Sec8 phosphorylation in the trafficking of GLUT4-containing vesicles has not clearly demonstrated in a previous study [44]; however, Sec5 phosphorylation was found to be critical for the dissociation of the exocyst complex from RalA, which then promotes the docking of GLUT4-containing vesicles to the plasma membrane [45]. The phosphorylation of Exo70 by EGF1 via ERK1/2 promotes the assembly of the exocyst complex in HEK293 cells [46]. Therefore, insulin as well as a subset of growth factors may regulate the assembly and disassembly of the exocyst complex through their downstream kinases. Moreover, Exo70 binds directly to Akt via TANK-binding kinase 1 (TBK1) [47]. TBK1 plays a key role in linking inflammation to insulin resistance in obesity [48,49]. As such, the exocyst-dependent pathway may play a significant role in regulating the metabolic crosstalk between glucose uptake, lipid uptake, and inflammation within adipocytes.

To conclude, our studies have demonstrated a novel role played by the exocyst complex in facilitating insulin-dependent FFA uptake by adipocytes. This newly identified biological pathway may help us better understand the molecular mechanism by which adipocytes regulate FFA handling in health and diseases.

## Supporting Information

S1 Fig Colocalization of Exo70 and lipid droplets in adipocytes. Intracellular localizations of mCherry-Exo70 and lipid droplets (BODIPY 493/503) were determined by confocal microscopy. Scale = 100 μm. In each sample, 3~4 cells were analyzed. Seven samples in total were used for colocalization analysis.
(TIFF)
