# Supplementary figures and images for: The Exocyst Complex Regulates Free Fatty Acid Uptake by Adipocytes
Source: PLoS One. 2015 Mar 13;10(3):e0120289. doi: 10.1371/journal.pone.0120289 (PMC4359155; doi:10.1371/journal.pone.0120289)

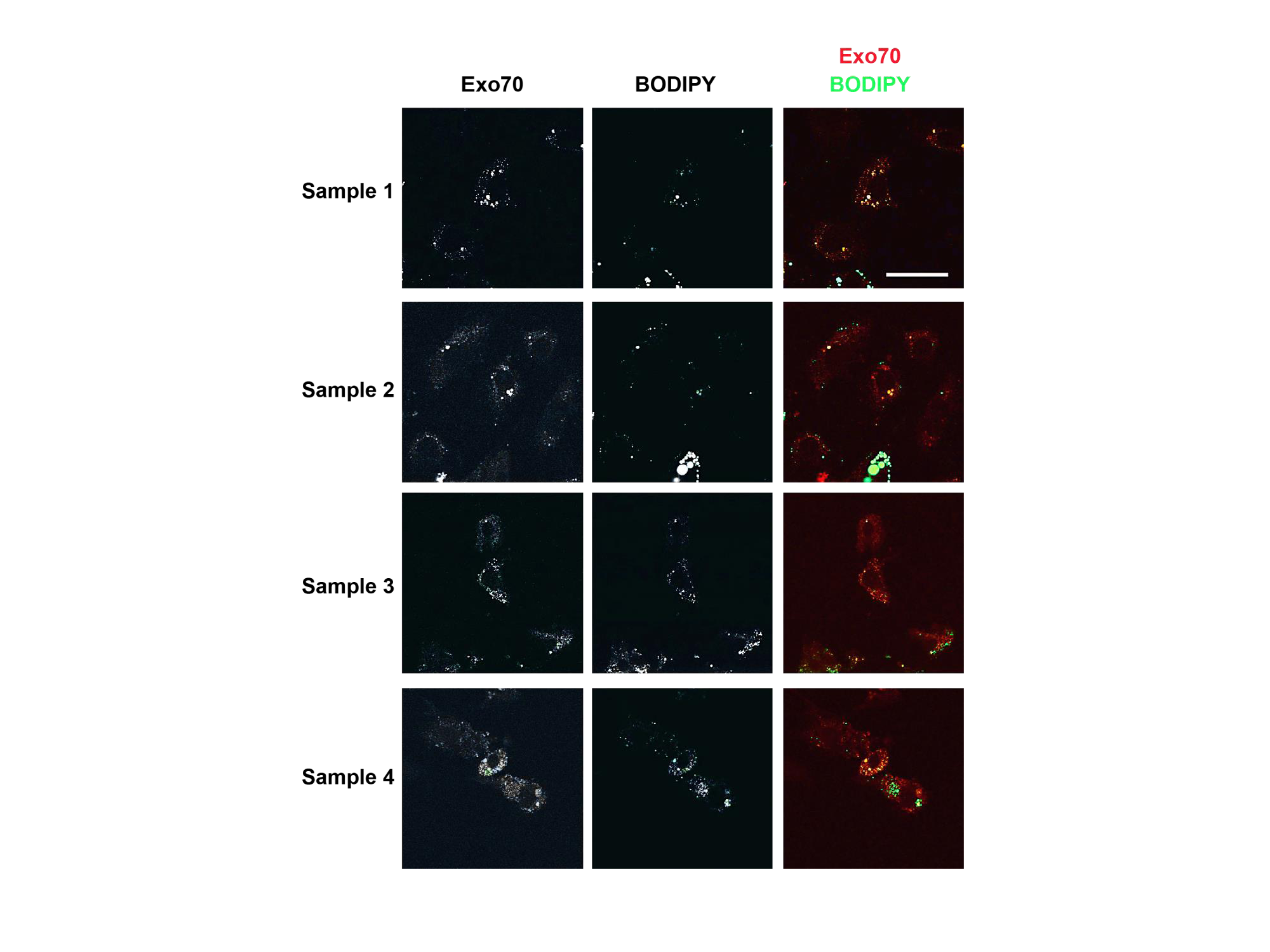

Supplement: S1 Fig — Intracellular localizations of mCherry-Exo70 and lipid droplets (BODIPY 493/503) were determined by confocal microscopy. Scale = 100 μm. In each sample, 3~4 cells were analyzed. Seven samples in total were used for colocalization analysis. (TIFF) [file pone.0120289.s001.tiff]
